# Current Overview of Treatment for Metastatic Bone Disease

**DOI:** 10.3390/curroncol28050290

**Published:** 2021-08-29

**Authors:** Shinji Tsukamoto, Akira Kido, Yasuhito Tanaka, Giancarlo Facchini, Giuliano Peta, Giuseppe Rossi, Andreas F. Mavrogenis

**Affiliations:** 1 Department of Orthopaedic Surgery, Nara Medical University, 840, Shijo-cho, Kashihara 634-8521, Nara, Japan; yatanaka@naramed-u.ac.jp; 2 Department of Rehabilitation Medicine, Nara Medical University, 840, Shijo-cho, Kashihara 634-8521, Nara, Japan; akirakid@naramed-u.ac.jp; 3 Department of Radiology and Interventional Radiology, IRCCS Istituto Ortopedico Rizzoli, Via Pupilli 1, 40136 Bologna, Italy; giancarlo.facchini@ior.it (G.F.); giuliano.peta@ior.it (G.P.); giuseppe.rossi@ior.it (G.R.); 4 First Department of Orthopaedics, School of Medicine, National and Kapodistrian University of Athens, 41 Ventouri Street, 15562 Athens, Greece; afm@otenet.gr

**Keywords:** metastasis, bone, cancer, nailing, resection, radiotherapy, chemotherapy, embolization, thermal ablation, electrochemotherapy

## Abstract

The number of patients with bone metastasis increases as medical management and surgery improve the overall survival of patients with cancer. Bone metastasis can cause skeletal complications, including bone pain, pathological fractures, spinal cord or nerve root compression, and hypercalcemia. Before initiation of treatment for bone metastasis, it is important to exclude primary bone malignancy, which would require a completely different therapeutic approach. It is essential to select surgical methods considering the patient’s prognosis, quality of life, postoperative function, and risk of postoperative complications. Therefore, bone metastasis treatment requires a multidisciplinary team approach, including radiologists, oncologists, and orthopedic surgeons. Recently, many novel palliative treatment options have emerged for bone metastases, such as stereotactic body radiation therapy, radiopharmaceuticals, vertebroplasty, minimally invasive spine stabilization with percutaneous pedicle screws, acetabuloplasty, embolization, thermal ablation techniques, electrochemotherapy, and high-intensity focused ultrasound. These techniques are beneficial for patients who may not benefit from surgery or radiotherapy.

## 1. Introduction

Bone metastases are a common complication of cancer and can be caused by most types of malignancies. They occur frequently in breast cancer (65–75%), prostate cancer (65–90%), and lung cancer (17–64%), and less frequently in thyroid cancer (65%), bladder cancer (40%), melanoma (14–45%), kidney cancer (20–25%), and colorectal cancer (10%) [1]. Bone lesions are found in 70–95% of multiple myeloma cases. It is estimated that there are approximately 280,000 new cases of bone metastases annually in the United States. As cancer treatment prolongs the overall survival of patients, the number of patients with bone metastases is expected to increase further [2]. Bone metastases cause pain, pathological fractures, compression of the spinal cord or nerve roots, and life-threatening hypercalcemia, and they may require surgery, radiotherapy, or medical treatment [3].

## 2. Diagnosis

Because recent weight loss may indicate that a malignant tumor has spread throughout the body, physicians need to ask patients if they have this symptom [4]. Patients should be asked about the symptoms associated with a particular type of primary cancer and the factors that cause primary cancer (e.g., smoking history, alcoholism, cirrhosis, hematuria, or abdominal pain) [4]. Physical examination may provide information about primary cancers that may cause bone metastases. Therefore, it is necessary to examine the breast, thyroid gland, skin, and lymph nodes, as well as perform a digital rectal examination and a urinalysis [4].

It is also necessary to determine whether a bone lesion is metastatic or primary. Primary bone malignancies must be ruled out as these require a completely different therapeutic approach [4]. Computed tomography (CT) or magnetic resonance imaging (MRI) may be useful for distinguishing between primary and metastatic bone tumors [5]. Soldatos et al. reported that compared with pathological fractures due to metastatic bone tumors, pathological fractures due to primary bone tumors more frequently had bone cortex lysis, calcification, and soft tissue mass on radiography, calcification, and soft tissue mass on CT, and periosteal reaction on MRI (*p* < 0.01) [5].

**Figure 1:**
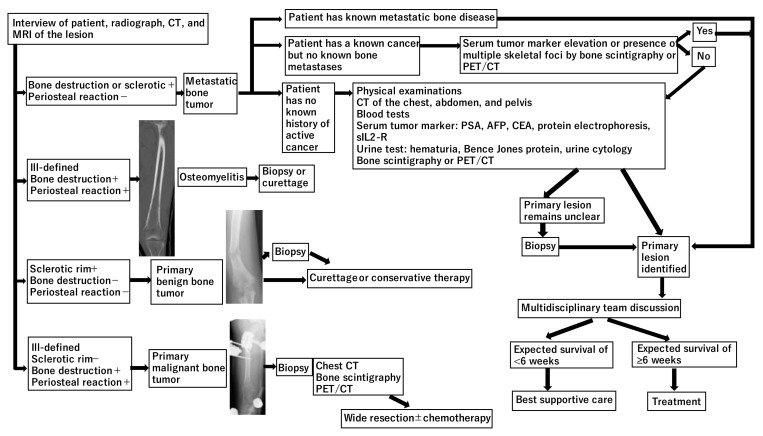
Diagnosis of bone metastases (PSA: prostate-specific antigen; AFP: α-fetoprotein; CEA: carcinoembryonic antigen; sIL2-R: soluble interleukin-2 receptor).

There are three main situations encountered in patients with bone metastases. If the patient has a known metastatic bone tumor, the new bone lesion is most likely a metastasis [4]. If the patient has a known cancer but no bone metastases, findings suggestive of metastases may include elevated serum tumor markers and the presence of multiple skeletal lesions on bone scintigraphy or ^18^F-fluorodeoxyglucose positron emission tomography (^18^F-FDG-PET/CT) [4]. Zhang et al. investigated 117 patients with a single known malignancy who underwent biopsy of a newly discovered bone lesion [6]. Of all the cases, 18% were benign lesions, and 3% were new malignancies. Patients with clinical symptoms were more likely to be diagnosed with bone metastases from known malignant tumors than patients without clinical symptoms (81% [87/107] vs. 50% [5/10]) [6]. For newly discovered bone lesions, a biopsy may be recommended in asymptomatic cases to avoid misdiagnosis; however, it should be avoided in symptomatic cases to reduce cost and risk [6]. If there is no history of active cancer or only a history of cancer in complete remission, the following tests should be performed: chest radiography, blood tests (calcium, albumin, alkaline phosphatase, blood cell counts, electrolytes, creatinine, erythrocyte sedimentation rate, C-reactive protein [CRP], and protein electrophoresis) [4]. If the above test results are normal, a CT scan of the chest, abdomen, and pelvis may be needed [4]. In addition, upper tract endoscopy, bronchoscopy, and serum tumor marker assays may be required based on CT findings [4]. A biopsy may be needed if the nature of the lesion remains unknown after the above tests [4]. A diagnosis algorithm may be useful to determine the approach and staging in any patient with a musculoskeletal lesion (Figure 1).

## 3. Prognosis

Physicians treating patients with bone metastases need to know the exact prognosis of the patient and weigh the benefits of surgery (improve function and control pain) against the risk of increased perioperative mortality [7]. A clinical factor-based prognostic scoring tool for patients with metastatic bone tumors has been developed to accurately predict life expectancy. Willeumier et al. created a prognostic model, the OPTModel, from the data of 1520 patients with bone metastases of long bones and who were treated with radiotherapy or surgery between 2000 and 2013 [8]. Patients were divided into four categories according to primary tumor, Karnofsky performance score, and the presence of visceral and/or brain metastases. Median survival was A: 21.9 months (95% confidence interval [CI], 18.7 to 25.1 months), B: 10.5 months (95% CI, 7.9 to 13.1 months), C: 4.6 months (95% CI, 3.9 to 5.3 months), and D: 2.2 months (95% CI, 1.8 to 2.6 months) for the 4 categories (Table 1 and Table 2) [8]. Another model is PathFx, a machine-learning Bayesian belief network applicable to patients with bone metastases in the trunk and limbs. The model includes both objective quantifiable variables (age, sex, primary type, Eastern Cooperative Oncology Group performance status score, presence of visceral metastases, presence of multiple skeletal metastases, pathological fracture, hemoglobin, and lymphocyte count), and subjective variables (surgeon’s estimate of survival), although it has been reported that prognosis can be accurately predicted without this subjective variable [9]. PathFx has been externally validated in many different patient populations [9,10,11,12] and has been recently updated to PathFx version 3.0 (https://www.pathfx.org accessed on 10 July 2021) [13]. Meares et al. [14] compared several models, including the revised Katagiri model [15], SSG score [16], Janssen nomogram [17], and SPRING 13 nomogram [18], and reported that OPTModel demonstrated the highest accuracy at predicting 12-month (area under the curve [AUC] = 0.79) and 24-month survival (AUC = 0.77) after surgical management, while PathFx was the most accurate at predicting 3-month (AUC = 0.70) and 6-month survival (AUC = 0.70). Similarly, Thio et al. successfully developed a machine-learning model that predicts 90-day and 1-year survival in patients with bone metastases in the extremities. The final model was incorporated into a freely accessible web application available at https://sorg-apps.shinyapps.io/extremitymetssurvival/ accessed on 10 July 2021 [19]. Table 3 summarizes the prognosis prediction model created by machine learning [9,10,11,12,13,14,19,20,21,22,23]. Errani et al. analyzed 159 patients with bone metastases in the extremities who underwent surgery [24] and reported that pathological CRP (≥1.0 mg/dL) and primary tumor diagnosis were significant negative prognostic factors at 12-month survival. Breast, kidney, prostate, and thyroid cancers were classified as good prognosis, while lung, unknown primary, liver, colorectal, bladder, pancreas, gastric, esophagus, testicle, tonsil cancers, sarcoma, and melanoma were classified as poor prognosis. The probability of survival at 12 months was 89% for group A: good prognosis primary tumor and physiological CRP; 57% for group B: bad prognosis primary tumor and physiological CRP or good prognosis primary tumor and pathological CRP; and 13% for group C: bad prognosis primary tumor and pathological CRP [24].

Karhade et al. reported that the 1-year survival rate was 58% in patients with alkaline phosphatase levels below 73 IU/L and 24% in patients with levels above 140 IU/L [25]. Kim et al. reported a significant increase in mortality in patients with non-small cell lung cancer with pathological fractures of the femur due to bone metastases [26]. In addition, mortality increased when bone metastases were located at the intertrochanteric area of the femur, decreased serum albumin levels increased mortality, and proper use of chemotherapy reduced the risk of death within 3 months after surgery [26]. Reconstruction with an endoprosthesis increased the risk of death, and elevated levels of serum leukocyte and alanine aminotransferase were associated with an increased risk of death within 1 month after surgery [26]. Sarahrudi et al. reported that pathological fractures of the upper limbs had a better prognosis than those of the lower limbs [27]. The median survival time of patients with pathological fractures of the humerus was 3.7 months (range: 0.2–71.2 months), while that of patients with pathological fractures of the femur was 2.7 months (range: 0.2–46 months) [27].

The main purpose of treating bone metastases is to improve symptoms and prevent the development of skeletal-related events. Surgical and/or medical treatment may be determined according to the prognosis of patients with cancer. Patients with a poor prognosis may be treated with less invasive palliative treatments [24,28]. Surgical treatment should be considered if life expectancy is expected to exceed 6 weeks. Patients with a life expectancy of 3–12 months should be treated with less invasive surgical reconstruction that does not require long-term rehabilitation. Patients with a life expectancy of more than 12 months should be treated with en bloc resection of bone metastatic lesions and durable reconstruction, such as megaprosthesis reconstruction, which requires long-term rehabilitation [24,28]. Resection of bone metastases is likely to prolong overall survival in patients who have renal cell carcinoma and solitary bone metastases [29]. Ruatta et al. retrospectively investigated 300 patients with bone metastases from renal cell carcinoma [29]. In multivariate analysis, conformant metastases were still predictors of poor prognosis; Memorial Sloan-Kettering Cancer Center risk group, radical resection, and synchronous solitary bone metastasis were predictors of better overall survival [29].

## 4. Bone Metastasis of the Extremities

The incidence of pathological fractures of the long bones due to bone metastases was 10% to 29% [30,31]. In 1989, Mirels reported a scoring system that predicted the risk of pathological fractures of the extremities [32]. It consisted of four components: the site of bone metastases, the degree of pain, radiographic findings, and lesion size. Each component was assigned a value from one to three. Therefore, total Mirels’ scores ranged from 4 to 12 (Table 4). He estimated that patients with a score of 9 had a 33% risk of fracture and suggested that surgical intervention should be considered if the score was 9 or higher [32]. However, the agreement rate between examiners for the Mirels’ score was moderate, and it reportedly lacked reproducibility in predicting the risk of pathological fractures [33]. Van der Linden et al. reported that only axial cortical involvement >30 mm (*p* = 0.01) and circumferential cortical involvement >50% (*p* = 0.03) were predictors of fractures due to femoral metastases [34]. Shinoda et al. retrospectively analyzed 161 bone metastases on CT images and suggested that surgery should be considered if medial cortical lesions in the proximal femur affected 25–50% of the entire circumference [35]. Philipp et al. retrospectively investigated 950 patients with femoral metastases, of whom 362 (38%) underwent prophylactic stabilization of femoral lesions and 588 (62%) underwent internal fixation after the occurrence of pathological fracture [36]. After adjustment for comorbidities and cancer types, patients who received prophylactic stabilization had a lower risk of death than those who underwent internal fixation after the occurrence of a pathological fracture [36]. Ward et al. and McLynn et al. reported that patients who underwent internal fixation after the occurrence of a pathological fracture had more intraoperative bleeding than those who underwent prophylactic stabilization of femoral metastases [37,38]. Thus, it seems important to predict pathological fracture due to metastases using the methods described above, as well as to perform prophylactic stabilization for patients with a high risk of pathological fracture.

En bloc resection and prosthetic reconstructions may be considered for bone metastases of the proximal femur, distal femur, and proximal humerus, especially in cases involving the metaepiphysis [24,28]. Intramedullary nails may be used for impending fractures and pathological fractures of the diaphysis. If there is a large bone defect, intramedullary nailing with curettage of bone metastases and bone cement filling may be indicated [24,28]. According to a systematic review of bone metastases in the extremities, the mechanical failure rate of intramedullary nails for pathological fractures of long bones due to bone metastases ranged from 2 to 22%, while that of megaprosthesis reconstruction was approximately 3.7% [28]. Bindels et al. retrospectively investigated 1090 patients with surgically treated long bone metastases [39]; 31% of patients developed complications within 1 month after surgery. Complications within 1 month post-operation were associated with increased 1-year mortality [39]. Tanaka et al. retrospectively analyzed 148 patients who underwent surgery for femoral metastases [40]. Risk classification was performed according to the Katagiri and revised Katagiri scoring systems [15,41]. In general, the low-risk group underwent resection and prosthetic reconstruction, and the high-risk group underwent internal fixation and radiotherapy. The choice of surgery in the medium-risk group depended on the patient’s condition, degree of bone destruction, and radiosensitivity. The 1-year survival rate was 71% in the resection and prosthetic reconstruction group and 15% in the internal fixation group (*p* < 0.001), and their 1-year local recurrence rates were 7% and 33%, respectively. The 2-year and 5-year local recurrence rates for the prosthetic reconstruction group were both 12%. Although the ambulatory rate was 99% for the prosthetic reconstruction group and 60% for the internal fixation group, the median time to ambulation was shorter in the internal fixation group (28 days vs. 23 days; *p* < 0.001) [40]. Erol et al. retrospectively investigated 115 patients who underwent en bloc resection, prosthetic reconstruction with or without cement fixation, and postoperative radiotherapy for long bone metastases [42]. There were 8 (6%) complications including aseptic loosening (*n* = 2), femoral stem breakage (*n* = 2), periprosthetic fracture (*n* = 2), and infection (*n* = 2). The frequency of complications was not significantly different between the cemented and non-cemented groups. The cemented group had significantly better Musculoskeletal Tumor Society functional scores [42]. Gainor et al. reported that bone union of pathological fractures occurred in 67% of fractures due to multiple myeloma, 44% of fractures due to renal cell carcinoma, and 37% of fractures due to breast cancer [43]. They reported that none of the patients with pathological fractures due to lung cancer had bone union before death [43]. Previous history of radiation may further reduce bone union. This may need to be considered when assessing the durability of intramedullary nails and plates preoperatively [43]. Thus, it may be vital to select surgical methods considering the patient’s prognosis, quality of life, post-operative function, and risk of post-operative complications.

Capanna et al. stated that single bone metastatic lesions located in the rib, clavicle, and expandable bones, such as the fibula and distal ulna, can be resected without functional loss [44]. They stated that the risk of mechanical failure associated with surgery for bone metastases around the elbows, knees, and ankles was low. If less than half of the bone is affected, they recommended cementing and plate fixation after curettage [44]. They also recommended local adjuvant therapy, such as phenol and cryotherapy, and radiotherapy after curettage [44]. If more than half of the metaphyseal region is affected, they recommended intra-articular resection and reconstruction with prosthesis for the distal humerus, distal femur, and proximal tibia, or arthrodesis with an allograft for ankle lesions [44].

Willeumier et al. examined the literature on the effects of adjuvant radiotherapy after surgical fixation for impending or pathological fractures of long bones. They found that there were only two retrospective cohort studies and the quality of evidence was very low [45]. It was not possible to conclude that postoperative radiotherapy after surgical stabilization was the standard treatment [45]. For patients with a long life expectancy, the benefits of radiotherapy may outweigh the disadvantages, but for patients with short life expectancy, the adverse effects of radiotherapy on quality of life may outweigh the risk of local progression or implant failure [45].

Metastases at the distal ends of the extremities (acrometastasis) account for approximately 0.1% of bone metastases [46,47,48,49,50]. This is usually due to lung cancer (>50%) or kidney cancer, followed by colon cancer, breast cancer, and genitourinary tract cancers [46,47,48,49,50]. This can occur in patients of all ages and tends to be slightly more common in men [46,48]. Patients with acrometastases generally have a poor prognosis due to widespread disease [51]. In 10% of the cases, acrometastasis may occur as the first presentation of occult cancer [52,53]. It can be mistaken for other benign diseases such as inflammatory lesions, cysts, gout, ganglia, osteomyelitis, tuberculous dactylitis, pyogenic granuloma, and primary skin tumors [48,54,55,56,57,58].

## 5. Bone Metastasis of the Spine

The spine is the most common site of metastatic bone tumors [59,60,61]. The most common site of spinal metastasis is the thoracic spine (60–70%), followed by the lumbosacral spine (20–25%) and the cervical spine (10–15%). Bone metastases to multiple spines occur in 17–30% of patients [59,60,61,62]. Metastatic spinal lesions can be osteolytic, osteoblastic, or mixed [63]. Constans et al. reported that more than 70% of spinal metastases are osteolytic lesions, 8% are osteoblastic, and 21% are mixed osteolytic and osteoblastic lesions [63]. Common primary cancers that cause osteolytic lesions are breast, lung, melanoma, renal, and thyroid cancers [64]. Primary cancers that cause osteoblastic lesions include carcinoids, medulloblastoma, nasopharyngeal cancer, prostate cancer, and urothelial cancer [64]. Breast, cervix, lung, and ovarian cancers can also cause a mixture of osteoblastic and osteolytic lesions [64].

Because at least 50% loss of mineral content and a minimum size of 1 cm on radiography are required to detect spinal metastases, it is often difficult to suspect early spinal metastases using radiography [65]. Bone scintigraphy may help detect spinal metastases due to its high sensitivity (62–100%) and specificity (78–100%) [66,67]. However, in the case of osteolytic or avascular lesions, bone scintigraphy may miss metastases [68,69]. In addition, degenerative changes may indicate an increase in tracer uptake on bone scintigraphy [70]. ^18^F-FDG-PET/CT had higher sensitivity (80–100%) and specificity (99%) in detecting spinal metastases than bone scintigraphy [66,71]. FDG-PET/CT definitely outperforms bone scintigraphy in osteolytic lesions [69,72,73,74,75]. However, FDG-PET/CT may not adequately evaluate osteoblastic metastases [72,76]. Both CT and MRI are essential for evaluating the characteristics of spinal metastasis. CT can distinguish between osteolytic and osteoblastic lesions and accurately assess lesions that affect the cortical bone [66,67]. CT can also be used to assess spinal stability according to the spinal instability neoplastic score (SINS) [77]. Whole-spine MRI can show detailed bone marrow findings and detect medullary metastases [3]. In addition, MRI can more clearly show the soft tissue and spinal cord [3]. Contrast enhancement can clearly show epidural extension [66]. MRI can detect spinal metastases earlier than other imaging modalities [78,79,80]. Spinal metastases often occur in the posterior vertebral body and spread to the pedicle [81]. MRI was recommended to consist of sagittal T1- and T2-weighted images of the entire spine and axial T2-weighted images of the affected spinal level [82,83]. Rades et al. investigated 2096 patients who received radiotherapy for metastatic spinal cord compression to determine the factors predicting ambulatory status after radiotherapy [84]. Multivariate analysis showed that five factors were significantly correlated with ambulatory status: primary tumor type, interval between tumor diagnosis and metastatic spinal cord compression, visceral metastases, motor function before radiotherapy, and time developing motor deficits before radiotherapy [84]. Therefore, because recovery from motor deficits may depend on the duration of symptoms and maintenance of ambulatory status, immediate MRI may be recommended if there is a possibility of metastatic epidural spinal cord compression or bilateral radiculopathy [84,85]. If the patient has a unilateral motor deficit and/or sensory deficit that indicates radiculopathy, MRI should be performed within 2–3 days [3]. MRI should be performed within 1 week for unilateral radiculopathy and within 2 weeks for localized pain [3].

Neurological considerations primarily focus on the degree of spinal cord compression. The 6-point epidural spinal cord compression (ESCC) grade may help determine the treatment of spinal cord metastases along with clinical assessment of myelopathy and/or radiculopathy [83,86]. ESCC grade is evaluated on axial T2-weighted images at the most severe compression sites. If the spine is stable, radiation may be considered for lesions with low-grade compression (ESCC grades 0, 1a, 1b, and 1c). For lesions with severe compression (grades 2 and 3), minimal surgical decompression of the epidural space may be considered unless the tumor is radiosensitive or the patient cannot tolerate surgery [86]. Breast and prostate cancer, lymphoma, seminoma, and myeloma are radiosensitive, while renal cell carcinoma, melanoma, and gastrointestinal tumors are radio-insensitive [87]. Surgery for spinal metastases due to radiation-sensitive tumors, such as multiple myeloma, malignant lymphoma, and leukemia should be avoided [88,89,90]. Quraishi et al. reported that Frankel grade was improved by surgical decompression in 17.5% of patients with lower-grade compression (ESCC grades 0, 1a, 1b, and 1c) and in 33% of patients with higher-grade compression grade (ESCC grades 2 and 3) [91]. Assessment of the degree of spinal cord compression may be important in determining whether separation surgery is needed to safely and effectively perform spinal stereotactic radiation therapy and radiosurgery [92]. Stereotactic radiotherapy for radio-insensitive tumors has shown higher local control rates than external beam radiotherapy [93,94,95]. Sohn et al. conducted a matched-pair study comparing the results of stereotactic radiosurgery and external beam radiotherapy when used as a first-line treatment for spinal metastases from renal cell carcinoma [94]. The decrease in perioperative visual analogue scale scores was greater in the stereotactic radiosurgery group than in the radiotherapy group (*p* = 0.04). Progression-free survival was significantly longer in the stereotactic radiosurgery group (*p* = 0.01) [94]. Treatment should be started within 24 h if there is metastatic spinal cord compression and neurological symptoms, within 3 days if there is metastatic spinal cord compression but no neurological symptoms, or within 2 weeks if there is only pain [3].

Surgical stabilization or percutaneous cement augmentation may be indicated for spinal instability, regardless of the ESCC grade and tumor radiosensitivity [86]. Radiotherapy may not improve spinal stability [86], defined as the “loss of spinal integrity due to a neoplastic process that is associated with movement-related pain, symptomatic or progressive deformity, and/or neural compromise under physiologic loads” [77]. The assessment of spinal instability is based on both clinical symptom criteria and CT imaging criteria. In 2010, the Spine Oncology Study Group created the SINS according to the best evidence provided by systematic review [77,96]. In this scoring system, spinal instability is assessed by adding five imaging and one clinical component score: spinal lesion level, presence and type of pain, lesion bone quality, spinal alignment, extent of vertebral body collapse, and posterolateral involvement of the spinal elements [77]. The total SINS ranges from 0 to 18 points and is divided into three clinical categories: 0–6 points suggests stability, 7–12 points suggests impending instability, and 13–18 points denotes instability (Table 5). Consultation with a spine surgeon may be recommended for patients with SINS ≥ 7. SINS showed excellent inter- and intra-observer reliability in determining spinal instability [97]. Its sensitivity and specificity were 96% and 80%, respectively [97]. However, there are some limitations to the SINS. For multiple spinal metastatic lesions, SINSs are not summed. Previous laminectomy or other surgical procedures and previous radiotherapy can affect spinal instability along with low bone quality, patient weight, and activity level [98].

Park et al. compared the outcome between patients with spinal metastases as the first symptom of malignancy and patients with prior treatment for primary malignancies who subsequently developed spinal metastases [99]. The survival time of the former group (mean 23 months) was significantly longer than that of the latter group (mean 15.5 months) [99].

A randomized, multi-institutional, non-blinded trial conducted by Patchell et al. in 2005 showed that direct decompressive surgery and postoperative radiotherapy were superior to radiotherapy alone in patients with metastatic spinal cord compression [88]. In this study, patients with metastatic spinal cord compression were randomly assigned to either surgery with subsequent radiotherapy (*n* = 50) or radiotherapy alone (*n* = 51). Significantly more patients were able to walk after treatment in the surgery with radiotherapy group (42/50, 84%) than in the radiotherapy alone group (29/51, 57%) (odds ratio 6.2 (95% CI 2.0–19.8), *p* = 0.001) [88]. Two subsequent meta-analyses confirmed that decompression surgery with postoperative radiotherapy resulted in a better ambulatory status than radiotherapy alone [100,101]. However, surgery is not always an option for patients with spinal metastases. Spine surgery should only be performed if the patient has a life expectancy of more than 6 months, and if clinical improvement outweighs the risks associated with surgery [61,102]. Pereira et al. identified the factors related to complications that occurred within 30 days of surgery for spinal metastases [103]. The surgical methods included corpectomy with stabilization (313 cases, 48%), decompression with stabilization (230 cases, 36%), decompression alone (84 cases, 13%), and stabilization alone (20 cases, 3.0%). Of the 647 patients, 205 (32%) had complications within 30 days [103]. Variables that were associated with these complications were lower albumin levels, additional comorbidities, pathologic fracture, three or more spinal levels operated on, and combined surgical approach. Complications within 30 days were associated with poor survival [103].

**Figure 2:**
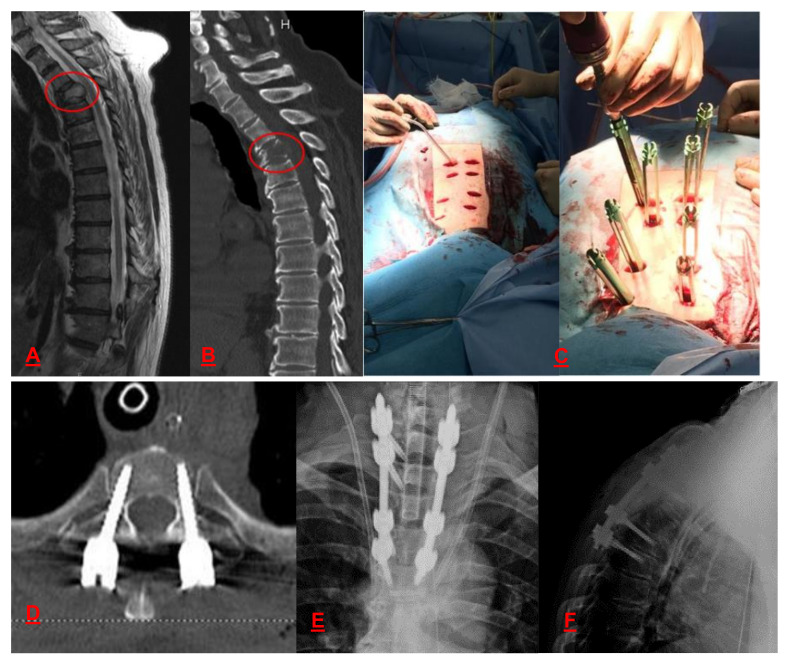
A 72-year-old man with T3 bone metastasis from prostate cancer treated with minimally invasive spine stabilization with percutaneous pedicle screws. (**A**) Sagittal T2-weighted magnetic resonance image shows a tumor bulging to the spinal canal. (**B**) Sagittal computed tomography (CT) shows a lytic lesion in the T3 vertebra. (**C**) Intra-operative photograph shows percutaneous pedicle screws osteosynthesis. Post-operative (**D**) axial, (**E**) anteroposterior, and (**F**) lateral CT radiographs show transpedicular screws osteosynthesis.

Less invasive spinal procedures include vertebroplasty, kyphoplasty, and minimally invasive spine stabilization with percutaneous pedicle screws. Vertebroplasty and kyphoplasty provide anterior column stability [104]. Vertebroplasty may relieve pain within 1-3 days [105]. Minimally invasive spine stabilization provides both anterior and posterior column stability (Figure 2) [106]. Therefore, these types of palliative surgery may be recommended for patients with a life expectancy of at least 3 months [3].

With regard to adjunct of spinal surgery, conventional spinal navigation solutions have been criticized for taking longer to set up and longer surgical time. However, augmented reality navigation solutions can simplify the setup and registration process using optical markers or surface recognition for patient tracking. Furthermore, it is possible to reduce the surgical time by obtaining the actual surgical field information and virtual information from the same field of view [107,108]. According to recent systematic review, with the augmented reality navigation solutions, the workflow was superior and the surgical accuracy was not inferior compared to the free-hand method or conventional navigation solutions [107]. In addition, the augmented reality navigation solutions reduced the radiation exposure of patients and staff [107]. Therefore, augmented reality navigation solutions may also be recommended for surgery of spinal metastasis.

When symptomatic metastatic spinal cord compression is identified, external beam radiotherapy and prompt administration of corticosteroids may be standard options, combined with supportive therapies, such as opioids, bisphosphonates, and spinal braces [109,110]. Embolization should also be considered for pain palliation in patients with spinal metastasis [111].

## 6. Bone Metastasis of the PELVIS

Bone metastases in the pelvis significantly impair the patient’s quality of life and require treatment [112,113,114,115,116,117,118,119]. Lesions in the ilium wing, sacroiliac joint, or anterior arch of the pelvis may have a low risk of mechanical failure and may usually be successfully treated with radiotherapy [44]. Surgery may be needed only if the patient has a good prognosis for solitary and late metastases at these sites [44]. In such cases, wide resection or aggressive curettage may improve patient survival [44]. The ilium wing and the anterior pelvic arch may cause less dysfunction after en bloc resection and may not require reconstruction other than reinforcement with synthetic mesh to avoid visceral hernias [44]. Periacetabular lesions are usually painful with weight bearing and are at risk of mechanical failure, resulting in progressive protrusio acetabuli. Therefore, surgical treatment and postoperative radiotherapy may be indicated to reduce pain, restore function, and allow early weight bearing [120]. If the medial wall of the acetabulum is involved, special cups with screwed lateral flanges may be used to prevent medial migration, and a metal mesh is placed on the medial wall before cementation. Different types of reinforcement rings may be used when both the medial and lateral walls of the acetabulum are destroyed. Harrington introduced another method using cement to fill the defect, which is reinforced by Steinmann pins placed into the pubic and ischial rami and from the acetabular dome to the sacroiliac joint [120]. This system improves the distribution of stress from the acetabular component to the spine and provides long-term durability of the implant [120]. Rowell et al. studied 46 patients who underwent acetabular cage and cement fixation to treat destructive acetabular metastases [121]. Four patients experienced postoperative dislocation, one experienced postoperative deep infection, and the other died within 30 days after surgery; 23 patients were able to walk independently, 10 used walking sticks, and 12 used walkers [121]. Tillman et al. studied 50 patients who underwent a modified Harrington procedure for periacetabular metastasis or hematological malignancy [122]. No perioperative deaths or complications occurred. Implant survival rates were 100% and 46% at 5 and 10 years, respectively. Ambulatory status and pain improved in 83% and 89% of patients, respectively [122]. They reported that the modified Harrington procedure for periacetabular metastasis had a lower incidence of complications, better functional outcomes, and reduced pain [122]. Houdek et al. compared the results of highly porous uncemented tantalum acetabular components (37 cases) and the modified Harrington procedure (78 cases). Eighty-three percent of the patients received adjuvant radiotherapy, and additional surgery was performed in 24 patients (21%). Harrington-style reconstructions had a higher reoperation rate than tantalum reconstructions (hazard ratio, 4.59; *p* = 0.003). Thirteen patients (11%) underwent acetabular component revision (11%); 5 (4%) were due to loosening of the acetabular component. The 10-year cumulative incidence acetabular component revision due to loosening was 9.6% in the Harrington-style reconstruction group and 0% in the tantalum reconstruction group (*p* = 0.09). After reconstruction, the mean Harris hip score improved significantly (31 to 67 points, *p* < 0.001), and there was no significant difference in the mean Harris hip score between the two groups (*p* = 0.29) [123]. Preoperative embolization may be recommended for all patients with pelvic bone metastases because all metastatic bone lesions are hypervascular and some are highly hypervascular [124].

Cotten et al. used the vertebroplasty technique to treat osteolytic metastatic lesions around the acetabulum [125]. Acetabuloplasty consisted of percutaneously injecting low-viscosity acrylic cement into the osteolytic cavity. The main goal is to increase the resistance of bone metastatic lesions to compressive stress and reduce the risk of fractures [126]. In addition, the exothermic reaction during cement polymerization may cause a local cytotoxic reaction to the tumor. Acetabuloplasty provided complete pain relief in 59% of patients [127]. Combining ablation treatment with cementoplasty may increase overall efficacy. In general, percutaneous treatment has a very low incidence of complications. Rare cases of hip cement protrusion without significant loss of function have been reported [128,129]. The indications for acetabuloplasty are pain and impending fractures [118,128,129]. Contraindications for acetabuloplasty include articular cortical destruction of the acetabular roof > 5 mm in diameter and soft tissue involvement more than three times the bone destruction area [118,128,129].

## 7. Radiotherapy

The main purpose of radiotherapy for bone metastases is to relieve pain, achieve local tumor control, and improve quality of life. After external beam radiotherapy, bone metastases usually begin to ossify within 3–6 weeks; partial response rates are up to approximately 60%, and complete response rates range from 10% to 25% [130,131,132,133]. The treatment for pain relief from uncomplicated bone metastases may be a single fraction of 8 Gy. Repeat radiotherapy may be effective in relieving symptoms that have recurred after single-fraction treatment [134]. An updated meta-analysis of palliative radiotherapy trials for bone metastases included 25 randomized controlled trials comparing single-fraction and multiple-fraction radiotherapy [135]. The overall and complete pain response rates were found to be equivalent. However, retreatment rates were 2.6 times higher for single-fraction radiotherapy [135]. Therefore, multiple-fraction radiotherapy that produce a sustained response may be recommended for patients with oligometastatic disease or good prognosis [136,137,138]. Stereotactic body radiation therapy is a highly concentrated form of radiation that can irradiate target tissue at a high dose without affecting the surrounding normal tissue. Although there is a high risk of vertebral compression fractures, it involves minimal toxicity to the spinal cord and has a high local control rate of spinal metastases [136]. A systematic review of complications of stereotactic body radiation therapy for spinal metastases found a 14% risk of vertebral compression fractures after stereotactic body radiation therapy [139]. In a retrospective study of 594 patients who underwent stereotactic body radiation therapy for spinal metastases, multivariate analysis revealed that a pre-existing vertebral compression fracture, a solitary metastasis, and a prescription dose of 38.4 Gy or higher increased the risk of compression fractures [140]. A meta-analysis of repeated radiation for painful bone metastases showed that repeated external beam radiation improved pain in only approximately 58% of patients [141], whereas repeated stereotactic body radiation therapy improved pain in 65–81% of patients [142].

Radiopharmaceuticals are currently used in patients with diffuse bone metastases, for which external beam radiation alone is not sufficient. Radionuclides, including beta-emitters such as Strontium-89 (^89^Sr) and Samarium-153 (^153^Sa), and alpha-emitters such as Radium-223 (^223^Ra) can be selectively delivered towards bone areas of amplified osteoblastic activity, sparing healthy organs from irradiation [143]. Beta-emitters can relieve pain but cause myelosuppression. The US Food and Drug Administration (FDA) has approved ^89^Sr and ^153^Sa radionuclides for the treatment of pain from bone metastasis, as well as ^223^Ra alpha-emitter for patients with castrate-resistant prostate cancer, symptomatic bone metastases and no known visceral metastatic disease [136]. In the placebo-controlled ALSYMPCA trial, ^223^Ra improved median overall survival in castrate-resistant prostate cancer patients with symptomatic bone metastases, compared with the control (15 vs. 11 months, *p* < 0.001), and prolonged the time to first skeletal-related event (16 vs. 10 months with placebo, hazard ratio 0.66, *p* < 0.001) [144]. However, recent meta-analyses showed no significant benefit in overall survival or symptomatic skeletal-related event-free survival in metastatic castration-resistant prostate cancer [145]. Patients need to have osteoblast activity in symptomatic bone metastases, with adequate renal function, bone marrow capacity, and life expectancy to receive radionuclide treatment [146]. In addition, radionuclide therapy is contraindicated in cases of spinal cord compression, a high risk of fracture of the bones of the lower extremities, or pregnancy and lactation [146].

## 8. Bisphosphonates and Denosumab

The histology of the primary tumor is the most important factor in choosing a chemotherapy regimen aimed at controlling tumor progression and preventing the development of skeletal-related events. Bisphosphonates indirectly reduce osteoclast activity by affecting osteoblasts, and directly induce osteoclast apoptosis by inhibiting farnesyl pyrophosphate synthase [1]. Among the intravenous agents, zoledronic acid is approved for the treatment of bone metastases in solid tumors and multiple myeloma, and pamidronate is approved for patients with breast cancer and multiple myeloma. Ibandronate can be administered both intravenously and orally and is effective for bone metastases in breast cancer patients. Oral clodronate is another treatment option for osteolytic bone metastases [147]. For breast cancer, bisphosphonates should be administered from the first presentation of bone metastases, even if they are asymptomatic. In prostate cancer, patients with hormone-sensitive disease have a lower risk of skeletal-related events, so bisphosphonates should only be administered to castrate-resistant patients. Bisphosphonates should be considered in patients with bone metastases from other malignancies who develop symptoms of skeletal-related events. Zoledronic acid is most effective in reducing serum calcium levels in patients with hypercalcemia, a serious and potentially life-threatening complication of osteolytic bone metastases [147]. Bisphosphonates have a risk of adverse events such as osteonecrosis of the jaw, renal failure, and hypocalcemia [147]. Zoledronic acid is usually administered every 3–4 weeks. However, a less intensive schedule (every 12 weeks) was reportedly non-inferior for breast cancer, prostate cancer, and multiple myeloma [148,149,150]. Therefore, a three-month schedule can reduce the risk of adverse events without affecting treatment outcomes.

Denosumab is a human monoclonal antibody that targets the receptor activator of NF-κB ligand (RANKL), a protein that acts as the primary signal to promote bone loss, inhibits the interaction between RANKL and RANK and, therefore, reduces osteoclast maturation and activity. The guidelines for denosumab for metastatic bone disease are similar to those for bisphosphonates. However, denosumab is not nephrotoxic and can be used in patients with renal failure. Hypocalcemia and osteonecrosis of the jaw are the most common complications of denosumab [134,147]. Currently, there is no evidence to support a reduced frequency of denosumab treatment; unlike bisphosphonates, denosumab does not accumulate in bone, even after months, and its interruption may compromise the therapeutic effect [151].

Lipton et al. performed a randomized controlled trial comparing denosumab and zoledronic acid for their ability to prevent skeletal-related events of bone metastases from various cancers [152]. Denosumab was superior to zoledronic acid in preventing skeletal-related events in patients with bone metastases, regardless of the Eastern Cooperative Oncology Group performance status, baseline visceral metastasis presence/absence, bone metastasis number, and urinary N-telopeptide level [152]. Chen et al. conducted a meta-analysis of six randomized controlled trials to compare the safety of denosumab and zoledronic acid for bone metastases [153]. Regarding minor adverse events, anemia and anorexia were more common in the zoledronic acid group, but the occurrence of back pain, nausea, fatigue, constipation, bone pain, arthralgia, and vomiting were not different between the two groups [153]. Regarding serious adverse events, there was no difference in osteonecrosis of the jaw between the two groups; however, renal adverse events were more common in the zoledronic acid group, and hypocalcemia and new primary malignancy were more common in the denosumab group [153].

## 9. Embolization

**Figure 3:**
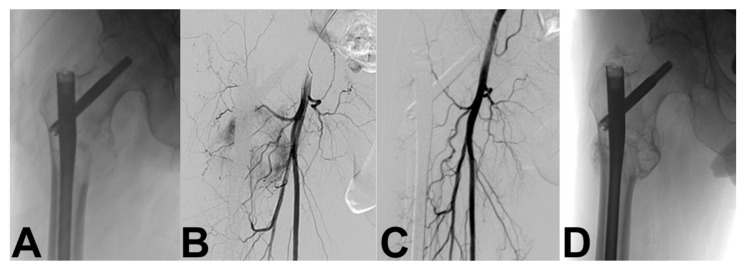
A 55-year-old man with right hip bone metastasis from renal cancer. (**A**) Prophylactic hip nail was done, (**B**) followed by digital subtraction angiography that showed the pathological tumor vessels, and (**C**) N-2-butyl cyano-acrylate selective embolization. (**D**) Post-embolization radiograph shows bone sclerosis (healing) of the bone metastasis without evidence of lesion progression at 6-month follow-up.

Embolization is a useful auxiliary procedure for bone metastases that can facilitate surgery by controlling bleeding, suppressing tumor growth, and relieving pain [154,155,156,157,158,159]. Most metastatic lesions are hypervascular; some lesions, such as renal (Figure 3) and thyroid metastases, are highly hypervascular [154,155]. Various embolic agents are currently available, including N-2-butyl cyano-acrylate, gelfoam, polyvinyl alcohol particles, alcohol emulsions, coils, tissue adhesives, ethanol, and microfibrillar collagen [52,134,160,161]. This procedure is technically successful if the intravascular contrast material completely stops the hypervascularity of the tumor, or if the vascularity of the tumor is reduced by more than 80% compared to the initial angiography [162].

Embolization of hypervascular bone metastases can reduce blood loss and surgical time, especially when performed on the day of surgery [163,164,165,166]. Complications that occur in 18–86% of embolizations are post-embolization syndromes that manifest as fever, pain, and malaise [162,167]. Other complications include neurologic complications, skin or muscle necrosis, and infections [154,167,168].

Rossi et al. performed 309 palliative embolizations with N-2-butyl cyano-acrylate in 243 patients with bone metastases. Fifty-six patients underwent repeated embolization at the same site within 1–3 months, while 197 underwent embolization for progressive lesions after radiotherapy. They reported a 50% or greater reduction in pain scores and analgesic needs in 97% of patients. The average duration of pain relief was 8 months (range: 1–12 months) [161].

## 10. Radiofrequency Thermal ABLATION (RFA)

RFA was reported to relieve the pain associated with bone metastases [169,170,171,172,173]. The Metastatic Spine Disease Multidisciplinary Working Group stated that RFA may be ineffective in treating bone metastases in the following situations: asymptomatic spinal metastases in patients with poor general condition, life expectancy of less than 6 months, pathological vertebral compression fracture, and epidural spinal cord compression [174]. Ablation methods, such as RFA or cryotherapy, are contraindicated if the tumor is within 1 cm of important structures such as the spinal cord, major nerves, and blood vessels [174].

Luigi Cazzato et al. performed RFA (*n* = 12; 25%) or cryotherapy (*n* = 37; 76%) in 49 patients with bone metastases, and the primary tumors were thyroid (*n* = 11, 23%), breast (*n* = 21; 43%), lung (*n* = 8; 16%), and other cancers (*n* = 9; 18%) [175]. Local progression at the treatment site was observed in 29% of cases (*n* = 14). The local control rates for 1 and 2 years were 77% and 72%, respectively. The local progression rate was higher when the size of the bone lesion was 2 cm or larger (*p* = 0.002) [175].

## 11. Electrochemotherapy

**Figure 4:**
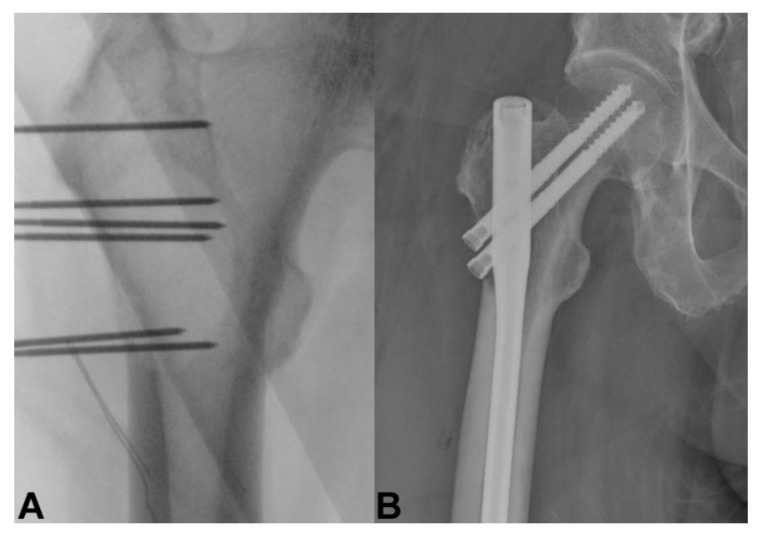
A 60-year-old man with right hip bone metastasis from thyroid cancer. (**A**) Electroporation and (**B**) prophylactic hip nailing was done without evidence of lesion progression at 6-month follow-up.

**Figure 5:**
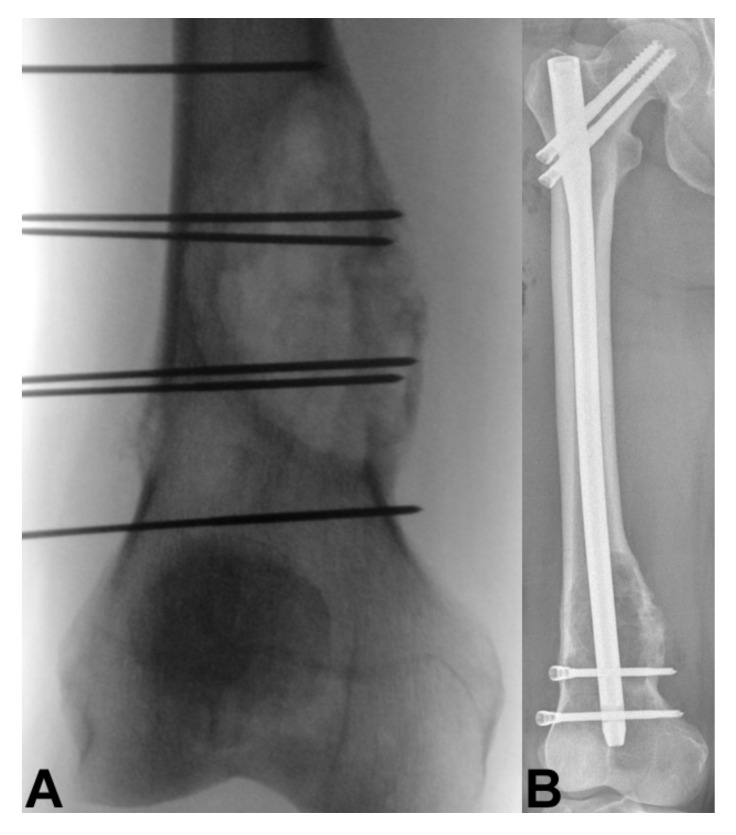
A 69-year-old man with right distal femur bone metastasis from renal cancer. (**A**) Electroporation and (**B**) prophylactic femoral nailing was done without evidence of lesion progression at 6-month follow-up.

Electrochemotherapy consists of a combination of electric pulses and intravenous infusion of chemotherapeutic drugs [176]. Cell membranes are usually poor or non-permeant, but electric pulses induce the opening of the transmembrane channels, which allows chemotherapeutic agents to enter the cell and enhance local cytotoxic effects [177,178]. Bleomycin and cisplatin have been shown to be the most effective and appropriate agents for electrochemotherapy in clinical use [134,179]. Recently, electrochemotherapy has become available for bone metastases (Figure 4 and Figure 5), improving pain relief and local control of bone metastases in patients who have failed radiotherapy or who have difficulty undergoing surgery [134]. The bone mineral structure was unchanged by electrochemotherapy, and the neural structure exhibited transient edema without structural changes after electroporation [180]. This is the main advantage of electrochemotherapy over radiotherapy and other ablation techniques [180,181]. Cornelis et al. reported that electrochemotherapy was performed in two patients with spinal metastasis and spinal cord compression to achieve pain relief, motor function improvement, and tumor growth control without complications, such as exacerbation of paralysis [182]. Campanacci et al. conducted a multicenter prospective study of 102 patients with bone metastases who underwent electrochemotherapy. Twenty-four patients (24%) received intramedullary nails scheduled after electrochemotherapy during the same surgery. Responses to treatment according to the Response Evaluation Criteria in Solid Tumors criteria were 40% objective responses, 51% stable disease, and 9% progressive disease. Breast cancer diagnosis and performance status of 0 to 1 were significantly associated with objective responses. During follow-up, a significant reduction in pain and a significant improvement in quality of life were observed [183].

## 12. High-Intensity Focused Ultrasound (HIFU)

HIFU causes coagulation necrosis at a thermal threshold of 65 °C to 85 °C, depending on the tissue absorption coefficient [184,185]. Ultrasound beam focus results in high intensities only at a specific location within a small volume that minimizes the potential for thermal damage to tissues outside the focal region [184,186,187,188]. MRI-guided focused ultrasound surgery (MRgFUS) combines high-intensity focused ultrasound with MRI guidance [189], and it has been approved for pain palliation in patients with bone metastases by the US FDA. In one study, MRgFUS relieved pain in 60–100% of patients; pain relief occurred rapidly and lasted for more than 3 months [190]. MRgFUS is recommended as a second-line treatment for pain relief in non-vertebral and non-skull metastases after radiotherapy failure. There are no restrictions based on the type of bone lesion (osteolytic or osteoblastic) or the number of bone lesions. Treatable lesions should always be identified on the image and restricted to the non-articular areas of the limbs, ribs, sternum, pelvis, shoulders, lumbar spine, and posterior sacrum. Further inclusion criteria are that lesions should be at least 10 mm below the skin surface, and the ultrasound beam path should always reach the target lesion without encountering other structures with high absorption or reflecting properties (such as non-target bone, air-filled organs, wide scars, or metallic implants/devices), as these shield the propagation of ultrasound and obscure targets beyond them [191].

Tsai et al. investigated factors correlated with therapeutic efficacy in 31 patients with bone metastases treated with MRgFUS. The overall clinical response rate was 84%, and the radiographic response rate was 68%. Multivariate analysis showed that good Karnofsky performance status and large lesion coverage volume factor (thermal ablative tumor volume/pretreatment tumor volume × 100%) correlated with a higher therapeutic effect [192].

According to the literature, the clinical response rate after MRgFUS ranges from 64% to 76% [193,194,195]. In one randomized controlled study, the MRgFUS group showed better response rates than the placebo group (64% vs. 20%, *p* < 0.001) [190]. The most common complication during MRgFUS treatment was pain (32%). Third-degree skin burns or fractures also occurred in 3% of patients [190]. Furthermore, a matched-pair design study compared MRgFUS and conventional radiotherapy in terms of efficacy as first-line local treatment, and MRgFUS had a higher response rate 1 week after treatment (71% vs. 26%, *p* = 0.0009) [195]. Randomized controlled trials from the Dutch Bone Metastasis Study database showed that MRgFUS was more effective when Karnofsky performance status was good and breast or prostate cancer was the primary cancer type [196]. Treatment of spinal metastases is not currently performed in clinical practice because of concerns about heat damage to the spinal cord [197].

## 13. Conclusions

The number of patients with bone metastasis is increasing as medical management and surgery improve the overall survival of patients with cancer. Bone metastasis can cause skeletal complications, including bone pain, pathological fractures, spinal cord or nerve root compression, and hypercalcemia. Before initiation of treatment for bone metastasis, it is important to exclude primary bone malignancy, which would require a completely different therapeutic approach. It is essential to select surgical methods and tools for patient prognosis, quality of life, postoperative function, and risk of postoperative complications. Therefore, the treatment of bone metastasis requires a multidisciplinary team approach by radiologists, oncologists, and orthopedic surgeons. Recently, many palliative treatments have emerged for bone metastases, such as radiotherapy techniques and radiopharmaceuticals, osteoplasties, minimally invasive spine stabilization with percutaneous pedicle screws, embolization, thermal ablation techniques, electrochemotherapy, and high-intensity focused ultrasound. These techniques are beneficial for patients who do not benefit from surgery or radiotherapy and are expected to increase further in the near future.

## Figures and Tables

**Table 1:** Prognostic model for survival estimation for patients with symptomatic long bone metastases (OPTModel) by Willeumier et al. [8].

Primary Tumor	Clinical Profile
Breast—positive ^1^	Favorable
Breast—unknown ^2^	Favorable
Kidney—solitary metastasis	Favorable
Thyroid	Favorable
Prostate	Moderate
Kidney—multiple metastases	Moderate
Soft tissue sarcoma	Moderate
Breast—triple-negative ^3^	Moderate
Kidney—unknown ^4^	Moderate
Endometrial carcinoma	Moderate
Osteosarcoma	Moderate
Ewing sarcoma	Moderate
Ovary	Moderate
Lung	Unfavorable
Colorectal	Unfavorable
Unknown primary	Unfavorable
Esophagus	Unfavorable
Bladder	Unfavorable
Melanoma	Unfavorable
Head and neck cancer	Unfavorable
Liver and/or pancreas	Unfavorable
Stomach	Unfavorable

^1^ Estrogen, progesterone, and HER2/neu positive. ^2^ Hormone receptor status and HER2/neu status were unknown. ^3^ Estrogen, progesterone, and HER2/neu negative. ^4^ The number of metastases was unknown.

**Table 2:** Prognostic model for estimating survival in patients with symptomatic long bone metastases (OPTModel) by Willeumier et al. [8].

Variables	Prediction of Prognosis											
1 Clinical profile	Favorable				Moderate				Unfavorable			
2 Karnofsky	80–100		≤70		80–100		≤70		80–100		≤70	
3 Visceral/brain metastases	No	Yes	No	Yes	No	Yes	No	Yes	No	Yes	No	Yes
Category	A	A	A	B	B	B	C	C	C	C	D	D
Median survival	21.9 months			10.5 months			4.6 months			2.2 months		

**Table 3:** Summary of the prognosis prediction models created by machine learning.

Model	Development	Variables to Input	Patient Population	Prediction of Survival	External Validation
PathFx model 1.0	Forsberg et al. [20], 2011	Not available	189 patients treated for metastatic bone disease between 1999 and 2003	Survival at 1, 3, 6, 12, 18, and 24 months	Forsberg et al. [10], 2012; Piccioli et al. [9], 2015; Forsberg et al. [12], 2017; Ogura et al. [11], 2017; Meares et al. [14], 2019
PathFx model 2.0	Overmann et al. [21], 2020	Not available	189 patients treated for metastatic bone disease between 1999 and 2003	Survival at 1, 3, 6, 12, 18, and 24 months	Overmann et al. [21], 2020
PathFx model 3.0 (https://www.pathfx.org)	Anderson et al. [13], 2020	Age, sex, oncologic diagnosis, pathological fracture, ECOG performance status, hemoglobin level, absolute lymphocyte count, number of skeletal metastases, organ metastases. In case of prostate cancer, PSA, patient age at metastases, treatment, race, comorbidity, alkaline phosphatase velocity.	208 patients treated for metastatic bone disease between 2015 and 2018	Survival at 1, 3, 6, 12, 18, and 24 months	Anderson et al. [13], 2020
Machine Learning Algorithm (https://sorg-apps.shinyapps.io/extremitymetssurvival/)	Thio et al. [19], 2020	Age, primary tumor type, visceral metastases, brain metastases, previous systemic therapy, hemoglobin level, white blood cell count, platelet count, absolute lymphocyte count, absolute neutrophil count, albumin level, alkaline phosphatase level, calcium, creatinine, sodium.	1090 patients treated surgically for long bone metastases between 1999 and 2017	Survival at 3 and 12 months	None
SORG Machine Learning Algorithm (https://sorg-apps.shinyapps.io/spinemets/, accessed on 10 July 2021)	Karhade et al. [22], 2018	White blood cell, albumin, American Society of Anesthesiologist Class, spine location, functional status, hematocrit, alkaline phosphatase.	1790 patients treated surgically for spinal metastatic disease between 2000 and 2016	Survival at 1 month	None
Machine Learning Algorithm (https://sorg-apps.shinyapps.io/spinemetssurvival/, accessed on 10 July 2021)	Karhade et al. [23], 2019	BMI, other Charlson comorbidity, primary tumor histology, ECOG performance status, ASIA, number of spine metastases, visceral metastases, brain metastases, previous systemic therapy, hemoglobin, platelet, absolute lymphocyte, albumin, alkaline phosphatase, creatinine, INR.	732 patients treated surgically for spinal metastatic disease between 2000 and 2016	Survival at 3 and 12 months	None

ECOG, Eastern Cooperative Oncology Group; PSA, Prostate Specific Antigen; BMI, Body Mass Index; ASIA, American Spinal Injury Association; INR, international normalized ratio.

**Table 4:** Mirels’ classification for impending pathological fractures [32].

Variable	Score		
	1 Point	2 Points	3 Points
Finding on imaging	Blastic	Mixed	Lytic
Size ^1^	<1/3	1/3–2/3	>2/3
Site	Upper extremity	Lower extremity	Peritrochanteric
Pain	Mild	Moderate	Mechanical

^1^ Size refers to the proportion of the diameter of the bone.

**Table 5:** Spinal Instability Neoplastic Score [77].

Variable	Score
**Location**	
Junctional (occiput–C2, C7–T2, T11–L1, L5–S1)	3
Mobile spine (C3–C6, L2–L4)	2
Semi-rigid (T3–T10)	1
Rigid (S2–S5)	0
**Pain**	
Yes	3
Occasional pain but not mechanical	1
Pain-free lesion	0
**Bone lesion**	
Lytic	2
Mixed (lytic/blastic)	1
Blastic	0
**Radiographic spinal alignment**	
Subluxation/translation present	4
De novo deformity (kyphosis/scoliosis)	2
Normal alignment	0
**Vertebral body collapse**	
>50% collapse	3
<50% collapse	2
No collapse with >50% body involved	1
None of the above	0
**Posterolateral involvement of spinal elements**	
Bilateral	3
Unilateral	1
None of the above	0
